# ^31^P-MRS, molecular level imaging for assessing muscular mitochondrial dysfunction in chronic kidney disease

**DOI:** 10.1093/ckj/sfae107

**Published:** 2024-04-08

**Authors:** Aurélie De Mul, Sandrine Lemoine

**Affiliations:** Service de néphrologie et d'exploration fonctionnelle rénale, Centre de référence des maladies rares du calcium et du phosphore, Centre de référence des maladies rénales rares, filières de santé maladies rares OSCAR, ORKID et ERKNet, hôpital Édouard-Herriot, hospices civils de Lyon, Lyon, France; Service de néphrologie et d'exploration fonctionnelle rénale, Centre de référence des maladies rares du calcium et du phosphore, Centre de référence des maladies rénales rares, filières de santé maladies rares OSCAR, ORKID et ERKNet, hôpital Édouard-Herriot, hospices civils de Lyon, Lyon, France

To the Editor,

We read with great interest the review titled ‘Making the invisible visible: imaging techniques for assessing muscle mass and muscle quality in chronic kidney disease’ by Alice Sabatino *et al*. [1] recently published in your journal. The review provides an insightful overview of the usefulness of imaging techniques to evaluate not only muscle quantity but also quality, particularly fat infiltration, in the context of chronic kidney disease (CKD) or end-stage kidney disease. The authors underline the importance of better understanding multifactorial muscle abnormalities, because they are predictive of worst outcomes. In this context, we would like to draw your attention to another functional imaging possibility: ^31^P magnetic resonance imaging spectroscopy (^31^P-MRS). ^31^P-MRS does not detect abundant proton signal like conventional resonance magnetic imaging. In contrast, signals from phosphorus-containing metabolites, such as phosphocreatine, inorganic phosphate and three different molecules of ATP—alpha, beta and gamma—are detected. This technique provides dynamic data on muscular oxidative metabolism and mitochondrial function by measuring intracellular phosphate and ATP variations *in vivo*, even during exercise and recovery. Mitochondrial function can be specifically evaluated by measuring phosphocreatine recovery time constant. Inflammation, oxidative stress, uremic toxin and myosteatosis can be associated with mitochondrial dysfunction in patients with CKD [2]. Limited data using ^31^P-MRS are available in the CKD population and would be beneficial to develop. Durozard *et al.* showed impaired muscular oxidative metabolism compensated by increased anaerobic glycolysis in dialyzed patients [3]. Comparing 21 controls subjects, 20 patients with CKD 3–5 and 22 patients on maintenance hemodialysis, Gamboa *et al.* demonstrated that muscular mitochondrial dysfunction was already present before initiation of dialysis [4]. Our group continuously monitored intracellular phosphate and ATP spectra during a 4-h hemodialysis session in 11 patients and revealed possible recurrent muscular ischemic injuries due to these metabolite depletions [5]. This exam should also be of greater interest to evaluate muscle metabolism in patients with CKD and tubulopathy with renal phosphate wasting.

Even if the technique availability may be limited by the requirement of a specific antenna and an experienced multidisciplinary team, this exam is non-invasive, not associated with radiation and does not require exogenous contrast. The leg can be scanned in isolation avoiding claustrophia and image acquisition time is reduced to <20 min. This technique can be combined with conventional magnetic resonance imaging. Due to its practicality, this exam can be easily performed and repeated, even in a patient undergoing dialysis [5] or exercising. It is particularly appealing for longitudinal monitoring or for recurrent assessment after a specific intervention such as an exercise program to see the impact of such initiatives on mitochondrial function. Therefore, ^31^P-MRS, an innovative imaging technique, may facilitate the translation of molecular-level oxidative metabolism abnormalities to a precise, *in vivo* evaluation of qualitative muscular dysfunction in CKD patient beyond low muscle mass.
